# Physical activity and recurrent fall risk in community-dwelling Japanese people aged 40–74 years: the Murakami cohort study

**DOI:** 10.1186/s11556-022-00300-5

**Published:** 2022-09-02

**Authors:** Shoto Kamimura, Takashi Iida, Yumi Watanabe, Kaori Kitamura, Keiko Kabasawa, Akemi Takahashi, Toshiko Saito, Ryosaku Kobayashi, Rieko Oshiki, Ribeka Takachi, Shoichiro Tsugane, Masayuki Iki, Ayako Sasaki, Osamu Yamazaki, Kei Watanabe, Kazutoshi Nakamura

**Affiliations:** 1 Niigata University School of Medicine, Niigata, Japan; 2 Division of Preventive Medicine, Niigata University Graduate School of Medical and Dental Sciences, Niigata, Japan; 3 Department of Health Promotion Medicine, Niigata University Graduate School of Medical and Dental Sciences, Niigata, Japan; 4 Department of Rehabilitation, Niigata University of Rehabilitation, Niigata, Japan; 5 Department of Health and Nutrition, Niigata University of Health and Welfare, Niigata, Japan; 6 Department of Food Science and Nutrition, Nara Women’s University Graduate School of Humanities and Sciences, Nara, Japan; 7 National Institute of Health and Nutrition, National Institutes of Biomedical Innovation, Health and Nutrition, Tokyo, Japan; 8 Department of Public Health, Faculty of Medicine, Kindai University, Osaka, Japan; 9 Murakami Public Health Center, Niigata, Japan; 10 Niigata Prefectural Government, Niigata, Japan; 11 Division of Orthopedic Surgery, Niigata University Graduate School of Medical and Dental Sciences, Niigata, Japan

**Keywords:** Cohort studies, Falls, Leisure activities, Physical activity, Risk factor

## Abstract

**Background:**

Falls are important causes of injury and mortality in older people, and associated medical costs can be enormous. Physical activity (PA) is a potential preventive factor for falls. However, few studies have examined the effect of different types of PA on fall prevention. This study aimed to evaluate the association between PA levels and the incidence of recurrent falls by type of PA in middle-aged and older people.

**Methods:**

This cohort study targeted 7,561 community-dwelling individuals aged 40–74 years who did not experience recurrent falls in the year before baseline. Information on PA levels, demographics, body size, lifestyle, and fall/disease history was obtained using a self-administered questionnaire in the baseline survey. Levels of total PA, leisure-time PA, and non-leisure-time PA (occupation, commuting, and housework) were estimated using metabolic equivalent (MET) scores (MET-h/day; hours spent on a given activity per day multiplied by its MET intensity). PA levels were categorized into four groups. Falls were recorded as none, once, or twice or more (recurrent falls). The outcome of the study was the incidence of recurrent falls in the past year before a survey conducted 5 years after the baseline survey. Logistic regression analyses were performed to calculate odds ratios for recurrent falls.

**Results:**

Higher total PA and non-leisure-time PA levels were associated with a higher risk of recurrent falls (*P* for trend = 0.0002 and 0.0001, respectively), with the highest total PA and non-leisure-time PA groups having a significantly higher adjusted OR (1.96 [95%CI:1.33–2.88] and 2.15 [95%CI:1.48–3.14], respectively) relative to the lowest group (reference). As for leisure-time PA, the medium group had a significantly lower adjusted OR (0.70 [95%CI:0.49–0.99]) relative to the reference group. By sex, the adjusted OR in the medium leisure-time PA group was significantly lower relative to the reference group in women (0.50 [95%CI: 0.29–0.85]) but not in men.

**Conclusions:**

Medium level leisure-time PA reduces the risk of recurrent falls in middle-aged and older people, whereas higher level non-leisure-time PA is associated with a higher risk of recurrent falls.

**Supplementary Information:**

The online version contains supplementary material available at 10.1186/s11556-022-00300-5.

## Introduction

Falls are an important risk factor for injuries and mortality [1]. Falls account for nearly 60% and 80% of all injuries in people aged 65–79 years and ≥ 80 years, respectively [2], and one third of fall injuries reportedly result in hospitalization and/or death [3]. Age-adjusted mortality from falls has increased in the last two decades [4]. Falls are also associated with enormous medical costs. Direct medical costs of falls in 2012 amounted to $616 million for fatal injuries and $30 billion for non-fatal injuries in the United States, which increased to $638 million and $31 billion, respectively, in 2015 [5]. According to the World Health Organization (WHO), the average health system cost per one fall injury episode for people aged ≥ 65 years in Finland and Australia (1999–2001) was US$ 3,611 [1]. In Japan, Hayashi [6] reported that medical and long-term care costs of falls were 730 million yen in 2002, which is equivalent to roughly 5% of the annual medical and long-term care costs spent by the population.

Regular physical activity (PA) has been shown to prevent non-communicable diseases such as heart disease, stroke, diabetes, and some cancers [7]. PA is also a potential preventive factor for falls. While intervention studies have shown that some types of exercise, such as balance and functional exercises (plus resistance exercises) and Tai Chi, are effective for fall reduction [8], the effect of PA in daily living on falls remains somewhat unclear. A recent meta-analysis found no association between PA levels and falls, and while individuals with lower PA levels had a significantly higher risk of recurrent falls (risk ratio = 1.4) than those with higher PA levels in two cohort studies, the relationship between PA levels and falls was inconclusive [9].

PA can be divided into four fundamental domains, including household, transportation, work, and leisure time [10, 11], and it is well established that leisure-time PA protects against mortality [12]. Leisure-time PA is also considered to be protective against functional limitations [13]. Some studies have examined the relationship between leisure-time PA levels and falls [14, 15], but few have examined other types of PA (non-leisure-time PA) and falls. Non-leisure-time PA, such as household activities, work-related transportation, and work, comprises much of our daily lives, and thus it is worth considering its relationship with falls.

We have been conducting a large-scale cohort study on musculoskeletal disorders (“Murakami cohort study”) since 2011 with the goal to identify risk factors for recurrent falls [16]. In the present study, we aimed to evaluate the association between levels of PAs, including total, leisure-time, and non-leisure-time PAs, and 5-year incidence of recurrent falls among middle-aged and older people.

## Methods

### Study design and participants

**Fig. 1:**
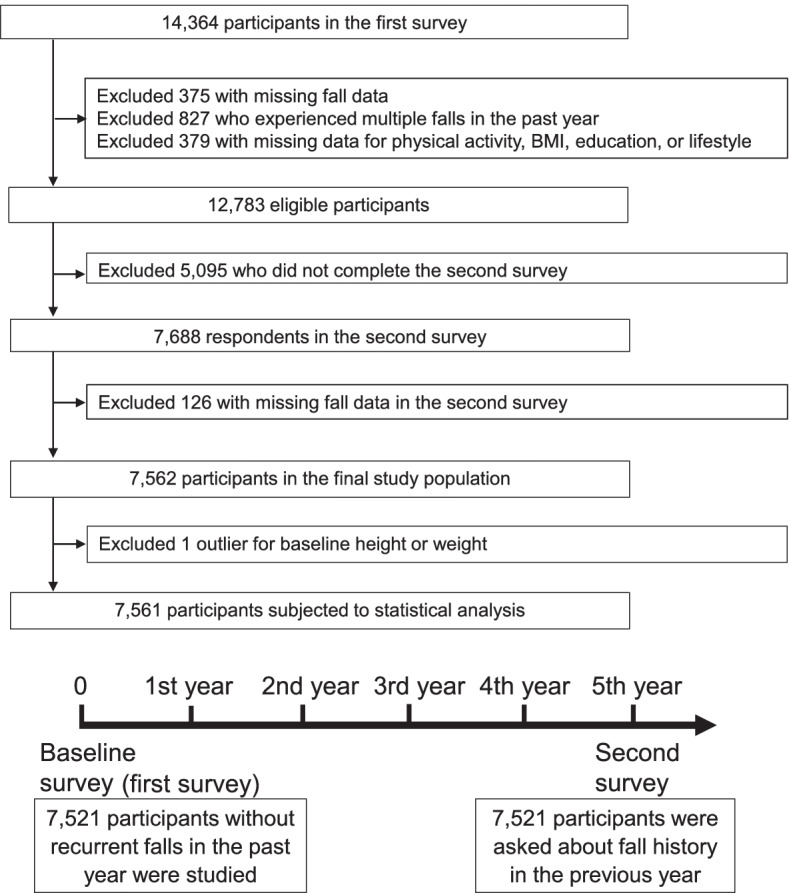
Flow chart of participant selection and structure of the study

This study used a cohort design. The predictor was PA levels in the baseline survey (hereafter referred to as the first survey), and the outcome was fall occurrence determined in a survey conducted 5 years after the baseline survey (hereafter referred to as the second survey). All 34,802 residents aged 40–74 years living in Murakami city, Sekikawa village, and Awashimaura village in Niigata Prefecture, Japan were originally recruited, and 14,364 completed the first survey. Details of participant selection and the structure of the study are outlined in Fig. 1. After excluding participants with missing data on falls, PA, body mass index (BMI), education, lifestyles, and multiple falls at baseline, the remaining 12,783 participants were considered eligible. Among the eligible participants, 7,688 (60.1%) completed the second survey, and 7,561 were included in the final study population after those with missing data on falls were excluded. All participants provided written informed consent. The study protocol was approved by the Ethics Committee of Niigata University School of Medicine (No. 2371). The protocol of the Murakami cohort study has been published elsewhere [16].

### Baseline survey (first survey)

The first survey was conducted in 2011–2013 using a self-administered questionnaire. The questionnaire elicited information on PA, sex, age, marital status, education, occupation, smoking habit, alcohol consumption, body height and weight, disease history (stroke, diabetes, and knee osteoarthritis), and falls.

The Japan Public Health Center-based prospective study-PA questionnaire (JPHC-PAQ) requested information on occupation-related activities (commuting, work, and housework) and leisure-time activities during free time in the previous year. Participants were asked to indicate the number of hours spent sitting, standing, walking, and doing strenuous work for occupational PA at different levels of intensity, as well as the frequency and number of hours spent walking slowly, walking quickly, performing light to moderate exercise (e.g., golf, croquet, and gardening), and performing strenuous exercise (e.g., tennis, jogging, aerobics, and swimming) for leisure-time PA at different levels of intensity. PA levels were estimated using metabolic equivalent (MET) scores (MET-h/day), which were calculated by multiplying the time spent on a given activity per day by its MET intensity. Total PA was classified into leisure-time PA (walking slowly, 2.8 METs; walking quickly, 4.0 METs; light-to-moderate exercise 3.0 METs; strenuous exercise, 6.0 METs), non-leisure-time PA (sitting, 1.3 METs; standing, 2.0 METs; walking, 3.0 METs; strenuous occupational work, 6.0 METs), sleep (0.9 METs), and other (1.3 METs). Spearman's correlation coefficients for activity volume (MET-hour/day) between JPHC-PAQ and 24-h activity records were reported to be 0.67 (*P* < 0.001) for total PA, 0.38 (*P* < 0.001) for sedentary behavior or light PA, 0.30 (*P* = 0.002) for moderate PA (0.002), 0.61 (*P* < 0.001) for moderate-to-vigorous PA, and -0.10 (*P* = 0.326) for vigorous PA, and their test–retest correlations were 0.48–0.74 (all *P* < 0.001), suggesting that JPHC-PAQ has acceptable validity and reliability for intensity-specific PA [17].

Marital status was classified as (a) married, (b) never married, and (c) divorced, separated, or bereaved. Education level was classified as (a) junior high school, (b) high school, (c) junior or vocational college, and (d) university or higher. Occupation was classified as (a) office work and sales/service work, (b) professional/management, (c) manual (security, farming/forestry/fishery, transportation, and labor services), and (d) no job/other. BMI was calculated as weight (kg) divided by height squared (m^2^). Correlation coefficients between self-reported and measured data were 0.985 for body height and 0.983 for body weight [12]. Smoking habit was classified as (1) non-smoker, (2) past smoker, (3) 1–20 cigarettes/day, and (4) ≥ 20 cigarettes/day. Alcohol consumption was classified as (1) non- or rare drinker, (2) 1–149 g ethanol/week, and (3) ≥ 150 g ethanol/week. History of fall (falling down to the ground from a standing position) in the year before the first survey was recorded as none, once, or twice or more (recurrent falls).

### Second survey

The second survey was conducted in 2016–2017 using a self-administered questionnaire. Completed questionnaire forms were collected via postal mail. The questionnaire solicited information on falls that occurred in the previous year in the same manner as in the first survey. Occurrence of recurrent falls (present or absent) in the past year was used as the outcome of this study because recurrent fallers are considered to be at risk of functional decline and mortality [18, 19]. The accuracy of fall recall in the past year was reported to be highly specific (91–95%) [20].

### Statistical methods

Participants with incorrect data, i.e., body height larger than 2.3 m or body weight larger than 200 kg, were excluded, resulting in a final analysis population of 7,561 participants. Mean values and standard deviations (SDs) were calculated for continuous variables. Statistical significance of linear associations between baseline characteristics and PA levels is presented with P for trend values calculated by simple linear or logistic regression analyses. The cumulative incidence of recurrent falls in the past year before the second survey was calculated. Simple and multiple logistic regression analyses were used to calculate P for trend values and odds ratios (ORs) to assess the effect of PAs on recurrent falls. Multiple logistic regression analysis was conducted by first adjusting for age (age-adjusted model); the model was further adjusted for basic potential confounders including demographic and lifestyle factors (Model 1), as well as for disease history (Model 2). Covariates in Model 1 included age, sex, marital status (dummy variable), education level, occupation (dummy variable), BMI, smoking habit, and alcohol consumption. Covariates in Model 2 included the history of stroke, diabetes, and knee osteoarthritis, in addition to Model 1 covariates. Non-leisure-time PA was also included as a covariate in multivariate analyses (both Models 1 and 2) for leisure-time PA and falls; leisure-time PA (dummy variable: medium level or others) was included as a covariate for non-leisure-time PA and falls. PA levels were categorized into four groups to compare ORs: levels of total PA and non-leisure PA were divided into quartiles (by sex), whereas levels of leisure-time PA were divided into “0” and tertiles (by sex) according to leisure-time PA MET scores (> 0), since more than 25% of participants had a MET score of 0. Subgroup analyses stratified by sex and age group (middle-aged: < 65 years; older: ≥ 65 years) were also conducted. ORs for falls were calculated according to intensity-based levels of leisure-time PA, as follows: 1) 0 min/week for both moderate- and strenuous-intensity exercises (reference), 2) low (moderate-intensity exercise for 0–149 min/week and strenuous-intensity exercise for 0–74 min/week [excluding 0 min/week for both exercises]), 3) moderate (moderate-intensity exercise for ≥ 150 min/week and strenuous-intensity exercise for < 75 min/week), and 4) high (strenuous-intensity exercise for ≥ 75 min/week), according to WHO recommendations (i.e., moderate-intensity exercise for ≥ 150 min/week and strenuous-intensity exercise for ≥ 75 min/week) [6], where “moderate-intensity exercise” included “walking quickly” and “light-to-moderate exercise” of JPHC-PAQ. The SAS (release 9.4, SAS Institute Inc., Cary, NC, USA) was used for statistical analyses. *P* < 0.05 was considered statistically significant.

## Results

First, we compared the baseline profiles of participants who did (completers) and did not (non-completers) complete the second survey. Completers (*n* = 7,688) had a higher mean age (60.9 vs 57.1 years, *P* < 0.0001 for men; 60.0 vs 57.0 years, *P* < 0.0001 for women) than non-completers (*n* = 5,095). Total PA (MET-h/day) levels did not differ between the two groups in both men (*P* = 0.1338) and women (*P* = 0.7258). Leisure-time PA levels were higher (2.08 vs 1.51 MET-h/day, *P* < 0.0001 for men; 2.11 vs 1.51 MET-h/day, *P* < 0.0001 for women), and non-leisure-time PA levels were lower (28.6 vs 29.9 MET-h/day, *P* = 0.0037 for men; 27.9 vs 28.8 MET-h/day, *P* = 0.0106 for women), in completers.

Among completers, mean ages of men, women, and men and women combined at baseline were 60.8 (SD 8.7), 59.8 (SD 8.8), and 60.3 years (SD 8.8), respectively. In men, 2,772, 506, and 166 experienced 0, 1, and ≥ 2 falls, respectively, and in women, 3,279, 678, and 142 experienced 0, 1, and ≥ 2 falls, respectively, in the past year before the second survey. The cumulative incidence of recurrent falls was 27/1,055 (2.6%) for those in their 40 s, 69/2,088 (3.3%) for those in their 50 s, 141/3,185 (4.4%) for those in their 60 s, and 71/1,233 (5.8%) for those in their 70 s. Participant characteristics at baseline according to levels of total PA, leisure-time PA, and non-leisure-time PA are shown in Table 1. Higher total PA levels were significantly associated with older age, higher leisure-time PA levels, higher non-leisure-time PA levels, lower education levels, a higher proportion of manual workers, a lower proportion of being jobless, and a higher proportion of those with a history of knee osteoarthritis. Higher leisure-time PA levels were significantly associated with older age, higher total PA levels, higher non-leisure-time PA levels, a lower proportion of manual workers, a higher proportion of being jobless, a lower proportion of smokers, and higher proportions of those with a history of diabetes and knee osteoarthritis. Higher non-leisure-time PA levels were significantly associated with older age, higher total PA levels, higher leisure-time PA levels, lower education levels, a higher proportion of manual workers, a lower proportion of being jobless, a higher proportion of smokers, and a higher proportion of those with a history of knee osteoarthritis.

**Table 1:** Participant characteristics according to four levels of each physical activity as measured by MET scores

	Quartiles of total PA (MET-hr/d)				*P* for trend
Characteristics	Q1 (*N* = 1,841)	Q2 (*N* = 1,929)	Q3 (*N* = 1,893)	Q4 (N = 1,898)	
Age^a^ (years)	57.8 (8.9)	60.0 (9.0)	61.3 (8.4)	61.9 (8.2)	< 0.0001
BMI^a^ (kg/m^2^)	23.4 (6.3)	23.1 (3.4)	23.3 (6.5)	23.2 (4.5)	0.7449
Total PA^a^ (MET-hr/d)	35.4 (1.5)	40.4 (1.6)	47.4 (2.9)	62.5 (7.0)	< 0.0001
Leisure-time PA^a^ (MET-hr/d)	0.7 (1.1)	1.8 (2.2)	2.7 (3.6)	3.0 (4.6)	< 0.0001
Non-leisure-time PA^a^ (MET-hr/d)	14.0 (4.6)	20.4 (5.1)	29.2 (6.5)	48.8 (10.3)	< 0.0001
Men	843 (45.8%)	874 (45.3%)	862 (45.5%)	865 (45.6%)	0.9381
Married	1,495 (81.2%)	1,611 (83.5%)	1,574 (83.1%)	1,536 (80.9%)	0.7357
University or higher	195 (10.6%)	170 (8.8%)	86 (4.5%)	32 (1.7%)	< 0.0001
Manual job	164 (8.9%)	248 (12.9%)	449 (23.7%)	752 (39.6%)	< 0.0001
No job	671 (36.5%)	894 (46.4%)	843 (44.5%)	618 (32.6%)	0.0054
Current smoker	277 (15.0%)	264 (13.7%)	296 (15.6%)	298 (15.7%)	0.2798
Current drinker	1,066 (57.9%)	1,114 (57.8%)	1,040 (54.9%)	1,054 (55.5%)	0.0523
Disease history					
Stroke	33 (1.8%)	48 (2.5%)	37 (2.0%)	30 (1.6%)	0.3987
Diabetes	157 (8.5%)	147 (7.6%)	153 (8.1%)	136 (7.2%)	0.1942
Knee osteoarthritis	130 (7.1%)	165 (8.6%)	189 (10.0%)	240 (12.6%)	< 0.0001
	Four levels of leisure-time PA (MET-hr/day)				*P* for trend
		Tertiles for scores > 0			
Characteristics	0 (N = 2,425)	Low (*N* = 1,679)	Medium (*N* = 1,711)	High (*N* = 1,746)	
Age^a^ (years)	57.4 (9.0)	59.2 (8.5)	61.2 (8.4)	64.4 (7.2)	< 0.0001
BMI^a^ (kg/m^2^)	23.3 (5.3)	23.3 (3.4)	23.1 (6.3)	23.2 (5.8)	0.3889
Total PA^a^ (MET-hr/d)	46.4 (11.9)	44.5 (10.7)	44.9 (9.8)	50.1 (9.8)	< 0.0001
Leisure-time PA^a^ (MET-hr/d)	0.0 (0.0)	0.4 (0.2)	1.8 (0.6)	6.9 (3.9)	< 0.0001
Non-leisure-time PA^a^ (MET-hr/d)	30.1 (16.4)	27.1 (15.0)	26.3 (13.8)	28.4 (12.8)	< 0.0001
Men	1,105 (45.6%)	763 (45.4%)	767 (44.8%)	809 (46.3%)	0.7700
Married	1,989 (82.0%)	1,395 (83.1%)	1,393 (81.4%)	1,439 (82.4%)	0.9620
University or higher	116 (4.8%)	142 (8.5%)	121 (7.1%)	104 (6.0%)	0.1483
Manual job	725 (29.9%)	343 (20.4%)	274 (16.0%)	271 (15.5%)	< 0.0001
No job	547 (22.6%)	554 (33.0%)	828 (48.4%)	1,097 (62.8%)	< 0.0001
Current smoker	518 (21.4%)	228 (13.6%)	223 (13.0%)	166 (9.5%)	< 0.0001
Current drinker	1,373 (56.6%)	981 (58.4%)	984 (57.5%)	936 (53.6%)	0.0796
Disease history					
Stroke	42 (1.7%)	31 (1.8%)	37 (2.2%)	38 (2.2%)	0.2330
Diabetes	139 (5.7%)	123 (7.3%)	146 (8.5%)	185 (10.6%)	< 0.0001
Knee osteoarthritis	175 (7.2%)	149 (8.9%)	186 (10.9%)	214 (12.3%)	< 0.0001
	Quartiles of non-leisure-time PA (MET-hr/d)				*P* for trend
Characteristics	Q1 (*N* = 1,852)	Q2 (*N* = 1,881)	Q3 (*N* = 1,929)	Q4 (*N* = 1,899)	
Age^a^ (years)	59.9 (8.9)	59.7 (9.0)	60.5 (8.8)	61.0 (8.3)	< 0.0001
BMI^a^ (kg/m^2^)	23.2 (6.2)	23.2 (3.4)	23.3 (5.7)	23.3 (5.5)	0.5269
Total PA^a^ (MET-hr/d)	36.5 (2.8)	40.4 (3.6)	46.9 (5.0)	61.9 (7.7)	< 0.0001
Leisure-time PA^a^ (MET-hr/d)	1.6 (2.6)	2.0 (2.9)	2.5 (3.8)	2.2 (3.7)	< 0.0001
Non-leisure-time PA^a^ (MET-hr/d)	12.5 (3.2)	20.4 (2.2)	29.7 (3.7)	49.7 (9.1)	< 0.0001
Men	828 (44.7%)	879 (46.7%)	869 (45.0%)	868 (45.7%)	0.8043
Married	1,511 (81.6%)	1,562 (83.0%)	1,612 (83.6%)	1,531 (80.6%)	0.5404
University or higher	174 (9.4%)	164 (8.7%)	116 (6.0%)	29 (1.5%)	< 0.0001
Manual job	152 (8.2%)	242 (12.9%)	458 (23.7%)	761 (40.1%)	< 0.0001
No job	851 (46.0%)	834 (44.3%)	790 (41.0%)	551 (29.0%)	< 0.0001
Current smoker	254 (13.7%)	286 (15.2%)	276 (14.3%)	319 (16.8%)	0.0237
Current drinker	1,043 (56.3%)	1,099 (58.4%)	1,073 (55.6%)	1,059 (55.8%)	0.3773
Disease history					
Stroke	45 (2.4%)	35 (1.9%)	36 (1.9%)	32 (1.7%)	0.1211
Diabetes	147 (7.9%)	173 (9.2%)	134 (6.9%)	139 (7.3%)	0.1355
Knee osteoarthritis	158 (8.5%)	162 (8.6%)	191 (9.9%)	213 (11.2%)	0.0021

Cut-off values are 37.8, 44.1, and 55.0 for men’s total PA quartiles; 37.8, 42.8, and 51.4 for women’s total PA quartiles; 0.9 and 3.0 for men’s leisure-time PA tertiles; 1.0 and 3.2 for women’s leisure-time PA tertiles; 15.9, 24.2, and 38.6 for men’s non-leisure-time PA quartiles; and 17.7, 24.6, and 36.1 for women’s non-leisure-time PA quartiles

*PA* physical activity

*BMI* body mass index, *MET* metabolic equivalent

^a^Mean with SD in parentheses

Cumulative incidence rates and ORs for recurrent falls according to levels of total PA, leisure-time PA, and non-leisure-time PA are shown in Table 2. Higher total PA levels were associated with a higher risk of recurrent falls (adjusted *P* for trend = 0.0002) in Model 2, with the highest total PA group (Q4) having a significantly higher adjusted OR (1.96) relative to the reference group (Q1). With respect to leisure-time PA, the medium group had a significantly lower adjusted OR (0.70) relative to the reference group (“0” group). However, the association between leisure-time PA levels and recurrent fall risk was not dose-dependent (adjusted *P* for trend = 0.2459 in Model 2).

**Table 2:** Odds ratios for recurrent falls according to four levels of each physical activity

	Quartiles of total PA (MET-hr/day)					*P* for trend
	Q1	Q2	Q3		Q4	
Cumulative incidence	50/1,841 (2.7%)	69/1,929 (3.6%)	80/1,893 (4.2%)		109/1,898 (5.7%)	
Unadjusted OR (95% CI)	1 (ref)	1.03 (0.99–1.07)	1.58 (1.10–2.26)		1.91 (1.35–2.71)	< 0.0001
Age-adjusted OR (95% CI)	1 (ref)	1.02 (0.99–1.06)	1.42 (0.99–2.05)		1.91 (1.35–2.71)	< 0.0001
Adjusted OR (1)^a^ (95% CI)	1 (ref)	1.02 (0.99–1.06)	1.46 (1.00–2.12)		1.89 (1.29–2.78)	0.0002
Adjusted OR (2)^b^ (95% CI)	1 (ref)	1.02 (0.99–1.06)	1.48 (1.01–2.16)		1.96 (1.33–2.88)	0.0002
	Four levels of leisure-time PA (MET-hr/day)					*P* for trend
		Tertiles for scores > 0				
	0	Low	Medium		High	
Cumulative incidence	106/2,425 (4.4%)	66/1,679 (3.9%)	57/1,711 (3.3%)		79/1,746 (4.5%)	
Unadjusted OR (95% CI)	1 (ref)	0.99 (0.96–1.02)	0.75 (0.54–1.05)		0.85 (0.61–1.17)	0.8342
Age-adjusted OR (95% CI)	1 (ref)	0.98 (0.95–1.02)	0.65 (0.46–0.90)		0.85 (0.61–1.17)	0.1056
Adjusted OR (1)^c^ (95% CI)	1 (ref)	0.99 (0.96–1.03)	0.71 (0.50–1.01)		0.90 (0.64–1.26)	0.3005
Adjusted OR (2)^b^ (95% CI)	1 (ref)	0.99 (0.96–1.03)	0.70 (0.49–0.99)		0.89 (0.63–1.25)	0.2459
	Quartiles of non-leisure-time PA (MET-hr/day)					*P* for trend
	Q1	Q2	Q3		Q4	
Cumulative incidence	51/1,852 (2.8%)	75/1,881 (4.0%)	69/1,929 (3.6%)		113/1,899 (6.0%)	
Unadjusted OR (95% CI)	1 (ref)	1.04 (1.00–1.08)	1.31 (0.91–1.89)		2.17 (1.55–3.04)	< 0.0001
Age-adjusted OR (95% CI)	1 (ref)	1.04 (1.00–1.08)	1.30 (0.90–1.87)		2.17 (1.55–3.04)	< 0.0001
Adjusted OR (1)^d^ (95% CI)	1 (ref)	1.04 (1.00–1.08)	1.20 (0.82–1.76)		2.13 (1.46–3.09)	0.0002
Adjusted OR (2)^b (^95% CI)	1 (ref)	1.04 (1.00–1.08)	1.22 (0.83–1.78)		2.14 (1.47–3.11)	0.0001

Cut-off values are 37.8, 44.1, and 55.0 for men’s total PA quartiles; 37.8, 42.8, and 51.4 for women’s total PA quartiles; 0.9 and 3.0 for men’s leisure-time PA tertiles; 1.0 and 3.2 for women’s leisure-time PA tertiles; 15.9, 24.2, and 38.6 for men’s non-leisure-time PA quartiles; and 17.7, 24.6, and 36.1 for women’s non-leisure-time PA quartiles

*OR* odds ratio, *PA* physical activity

^a^Adjusted for sex, age, marital status, education, occupation, body mass index, smoking habit, and alcohol consumption

^b^Further adjusted for disease history in addition to covariates in Model 1

^c^Adjusted for sex, age, marital status, education, occupation, body mass index, smoking habit, alcohol consumption, and non-leisure-time PA

^d^Adjusted for sex, age, marital status, education, occupation, body mass index, smoking habit, alcohol consumption, and leisure-time PA

Subgroup analyses showed that the medium group of leisure-time PA had a significantly lower adjusted OR (0.50, 95%CI:0.29–0.85) relative to the reference group (“0” group) in women, but not in men. The medium group also had a significantly lower adjusted OR (0.56, 95%CI:0.34–0.91) relative to the reference group (“0” group) in the < 65-year age group, but not in the ≥ 65-year age group. Higher non-leisure-time PA levels were associated with a higher recurrent fall risk (adjusted *P* for trend = 0.0001) in Model 2, with the highest non-leisure-time PA group (Q4) having a significantly higher adjusted OR (2.14) relative to the reference group (Q1). Cumulative incidence rates and ORs for recurrent falls according to levels of total PA, leisure-time PA, and non-leisure-time PA by sex, age group, and non-leisure-time PA levels are shown in Additional Tables 1, 2, and 3, respectively.

With respect to intensity-based levels of leisure-time PA, the high intensity group (i.e., strenuous-intensity exercise for ≥ 75 min/week) had a marginally significantly lower OR (0.61, 95%CI:0.34–1.08, *P* = 0.0909) for recurrent falls relative to the reference group (0 min/week); no significant differences were observed for other groups (Additional Table 4).

## Discussion

The present cohort study first evaluated the risk of falls by type of PA (leisure-time and non-leisure-time PAs), and found that medium leisure-time PA levels were associated with a lower risk of recurrent falls, whereas high leisure-time PA levels were not, suggesting that the association between leisure-time PA levels and fall risk is U-shaped. However, higher non-leisure-time PA levels were associated with a higher risk of recurrent falls. In addition, the association between medium leisure-time PA levels and a lower risk of recurrent falls was more robust in women than in men, and in middle-aged people than in older people.

In the present study, leisure-time PA levels were associated with a significantly reduced risk of recurrent falls in the medium group, possibly due to improved physical function. This finding is in line with previous studies reporting that exercise reduces fall risk in older people [8], and also supports a cohort study that suggested an association between moderate (and higher) PA levels and reduced fall risk [21]. However, we observed no decrease in the risk of recurrent falls in the high leisure-time PA group, suggesting that the association between leisure-time PA levels and fall risk is U-shaped. This finding is supported by a recently conducted accelerometer-based study, showing that the association between bouted activity levels and fall risk was U-shaped, but that of sporadic activity levels was not [22]. The preventive effect of leisure-time activity or exercise may be offset by an increased fall probability in active people.

In the present study, higher total PA levels were associated with a higher risk of recurrent falls, regardless of sex and age group. Previous cohort studies have reported similar findings [14, 23, 24]. A positive association between total PA levels and fall risk was observed in 5,995 men aged ≥ 65 years over a period of 4.5 years (OR of Q4 [v.s. Q1] = 1.2) [14] and 75,428 women aged 50–79 years over a period of 6 years (OR of Q4 [v.s. Q1] = 1.2) in the United States [23]. These findings (including the present findings) suggest that active people are more prone to falls. Compared to the two previous studies in the United States, however, the impact of total PA on fall risk in the present study appeared to be larger (OR of Q4 [v.s. Q1] = 1.9), i.e., the association between total PA levels and fall risk was stronger in our study population. The reason for this is unclear but may be related to differences in PA levels and/or fall rate. Lewis et al. [24] reported that high PA levels are associated with an increased risk of falls, particularly in people who have low levels of physical function. The findings of these studies, including ours, suggest that PA is potentially harmful, especially for older people.

Similar to total PA levels, non-leisure-time PA levels were also associated with recurrent fall risk. It should be noted, however, that ORs were generally higher for non-leisure-time PA than for total PA, and thus, the results regarding total PA may mainly reflect those of non-leisure-time PA.

The validity of JPHC-PAQ is generally acceptable, except that its accuracy when used for vigorous PA is poor. According to Kikuchi et al. [17], this is due to the overestimation of work-related vigorous PA. For this reason, there may have been misclassification of non-leisure-time PA groups. This is a limitation of the present study.

In the present study, medium leisure-time PA levels (1.0–3.0 MET-h/day for men and 1.0–3.4 MET-h/day for women) were associated with the occurrence of falls. These PA levels are nearly equivalent to 20–60 min/day (or 140–420 min/week) of “walking for pleasure” (3.5 METs) [25]. Medium leisure-time PA levels were set in accordance with the WHO recommendation of performing at least 150 min of moderate-intensity (3–5 METs) PA throughout the week [26]. As for leisure-time PA intensity, the high intensity group had a significantly lower risk of recurrent falls, suggesting that higher-intensity leisure-time exercise may be more effective in preventing falls.

The association between medium leisure-time PA levels and reduced risk of recurrent falls was more robust in women than in men in the present study. Since women are more likely to be sarcopenic and have limitations in functional activities than men [27], leisure-time PA may be more important for women in terms of maintaining their physical function, and hence, preventing falls. Moreover, estrogen, which has a protective effect on skeletal muscle [27], may be involved in the U-shaped association observed between leisure-time PA levels and fall risk in women. For example, moderate leisure-time PA levels are reportedly associated with high estrogen levels provided potentially by endogenous or exogenous factors [28].

There are several limitations in this study. First, we obtained information on PAs and falls from participant self-reports. Thus, PA data were subjective rather than objective. Future studies using objective PA data from a large cohort would be informative. We also collected retrospective fall occurrence data by self-report, and did not collect fall data for the period of 5 years from the first survey to the second survey, but instead used the information on falls during the past year before the second survey to determine the incidence of recurrent falls. Collectively, these limitations might have resulted in misclassification bias. In addition, we did not have information on other potential risk factors for recurrent falls, such as adverse events (e.g., injuries), during the 5-year period. A follow-up study to assess PA and falls annually may help clarify whether the progress in PA levels in the past year is a predictor of recurrent falls. Second, fall data were only obtained from participants who completed the second survey, and those who did not may have had a physical disability. This may have led to another bias that resulted in underestimation of ORs. Third, the proportion of participants engaging in walking quickly or light-to-moderate exercise at least once a week was 39%, whereas that for strenuous exercise was only 8% (data not shown), indicating that balance-type exercises were much more common than strength or endurance exercises. Therefore, our findings may not be generalizable to populations with a different exercise structure. Finally, we targeted community-dwelling people, most of whom were considered physically independent. Therefore, our results may not be generalizable to frail people who are likely institutionalized.

## Conclusions

In conclusion, medium leisure-time PA levels are associated with the risk of recurrent falls in middle-aged and older people, but high leisure-time PA levels are not, suggesting that the association between leisure-time PA levels and the risk of recurrent falls is U-shaped. Additionally, higher level non-leisure-time PA is associated with a higher risk of recurrent falls, suggesting that PA is potentially harmful. Further cohort studies are necessary to confirm our results.

## Supplementary Information

**Additional file 1: Table 1.** Odds ratios for recurrent falls according to four levels of each physical activity stratified by sex. **Table 2.** Odds ratios for recurrent falls according to four levels of each physical activity stratified by age group. **Table 3.** Odds ratios for recurrent falls according to levels of leisure-time PA by non-leisure-time PA subgroup. **Table 4.** Odds ratios for recurrent falls according to intensity-based levels of leisure-time physical activity.

## Data Availability

The datasets generated and/or analyzed during the current study are not publicly available, because participants did not consent to having their data provided to anyone outside the research group. However, the minimal dataset may be available upon ethical approval by the Ethics Committee of Niigata University. Please contact the Office of the Murakami Cohort Study (kazun@med.niigata-u.ac.jp) for inquiries about data availability.

## Abbreviations

PA: Physical Activity
BMI: Body Mass Index
OR: Odds Ratio
MET: Metabolic Equivalent
Q: Quartile
SD: Standard Deviation
WHO: World Health Organization
