# Correction: Physical activity and recurrent fall risk in community-dwelling Japanese people aged 40–74 years: the Murakami cohort study

**DOI:** 10.1186/s11556-022-00306-z

**Published:** 2022-10-24

**Authors:** Shoto Kamimura, Takashi Iida, Yumi Watanabe, Kaori Kitamura, Keiko Kabasawa, Akemi Takahashi, Toshiko Saito, Ryosaku Kobayashi, Rieko Oshiki, Ribeka Takachi, Shoichiro Tsugane, Masayuki Iki, Ayako Sasaki, Osamu Yamazaki, Kei Watanabe, Kazutoshi Nakamura

**Affiliations:** 1 Niigata University School of Medicine, Niigata, Japan; 2 Division of Preventive Medicine, Niigata University Graduate School of Medical and Dental Sciences, Niigata, Japan; 3 Department of Health Promotion Medicine, Niigata University Graduate School of Medical and Dental Sciences, Niigata, Japan; 4 Department of Rehabilitation, Niigata University of Rehabilitation, Niigata, Japan; 5 Department of Health and Nutrition, Niigata University of Health and Welfare, Niigata, Japan; 6 Department of Food Science and Nutrition, Nara Women’s University Graduate School of Humanities and Sciences, Nara, Japan; 7 National Institute of Health and Nutrition, National Institutes of Biomedical Innovation, Health and Nutrition, Tokyo, Japan; 8 Department of Public Health, Faculty of Medicine, Kindai University, Osaka, Japan; 9 Murakami Public Health Center, Niigata, Japan; 10 Niigata Prefectural Government, Niigata, Japan; 11 Division of Orthopedic Surgery, Niigata University Graduate School of Medical and Dental Sciences, Niigata, Japan

**Correction: Eur Rev Aging Phys Act 19, 20 (2022)**

**https://doi.org/10.1186/s11556-022-00300-5**

In the publication of the original article [1], numerical errors were found in the odds ratios in column Q2 of Table 2.

See below for the corrected Table 2.

**Table 2:** Odds ratios for recurrent falls according to four levels of each physical activity

	Quartiles of total PA (MET-hr/day)				*P* for trend
	Q1	Q2	Q3	Q4	
Cumulative incidence	50/1,841 (2.7%)	69/1,929 (3.6%)	80/1,893 (4.2%)	109/1,898 (5.7%)	
Unadjusted OR (95% CI)	1 (ref)	1.33 (0.92-1.92)	1.58 (1.10-2.26)	1.91 (1.35-2.71)	<0.0001
Age-adjusted OR (95% CI)	1 (ref)	1.26 (0.87-1.83)	1.42 (0.99-2.05)	1.91 (1.35-2.71)	<0.0001
Adjusted OR (1)^a^ (95% CI)	1 (ref)	1.25 (0.86-1.82)	1.46 (1.00-2.12)	1.89 (1.29-2.78)	0.0002
Adjusted OR (2)^b^ (95% CI)	1 (ref)	1.26 (0.86-1.84)	1.48 (1.01-2.16)	1.96 (1.33-2.88)	0.0002
	Four levels of leisure-time PA (MET-hr/day)				*P* for trend
		Tertiles for scores > 0			
	0	Low	Medium	High	
Cumulative incidence	106/2,425 (4.4%)	66/1,679 (3.9%)	57/1,711 (3.3%)	79/1,746 (4.5%)	
Unadjusted OR (95% CI)	1 (ref)	0.90 (0.65-1.23)	0.75 (0.54-1.05)	0.85 (0.61-1.17)	0.8342
Age-adjusted OR (95% CI)	1 (ref)	0.85 (0.62-1.16)	0.65 (0.46-0.90)	0.85 (0.61-1.17)	0.1056
Adjusted OR (1)^c^ (95% CI)	1 (ref)	0.94 (0.68-1.29)	0.71 (0.50-1.01)	0.90 (0.64-1.26)	0.3005
Adjusted OR (2)^b^ (95% CI)	1 (ref)	0.92 (0.67-1.28)	0.70 (0.49-0.99)	0.89 (0.63-1.25)	0.2459
	Quartiles of non-leisure-time PA (MET-hr/day)				*P* for trend
	Q1	Q2	Q3	Q4	
Cumulative incidence	51/1,852 (2.8%)	75/1,881 (4.0%)	69/1,929 (3.6%)	113/1,899 (6.0%)	
Unadjusted OR (95% CI)	1 (ref)	1.47 (1.02-2.11)	1.31 (0.91-1.89)	2.17 (1.55-3.04)	<0.0001
Age-adjusted OR (95% CI)	1 (ref)	1.48 (1.03-2.12)	1.30 (0.90-1.87)	2.17 (1.55-3.04)	<0.0001
Adjusted OR (1)^d^ (95% CI)	1 (ref)	1.43 (0.99-2.06)	1.20 (0.82-1.76)	2.13 (1.46-3.09)	0.0002
Adjusted OR (2)^b (^95% CI)	1 (ref)	1.44 (0.999-2.08)	1.22 (0.83-1.78)	2.14 (1.47-3.11)	0.0001

Cut-off values are 37.8, 44.1, and 55.0 for men’s total PA quartiles; 37.8, 42.8, and 51.4 for women’s total PA quartiles; 0.9 and 3.0 for men’s leisure-time PA tertiles; 1.0 and 3.2 for women’s leisure-time PA tertiles; 15.9, 24.2, and 38.6 for men’s non-leisure-time PA quartiles; and 17.7, 24.6, and 36.1 for women’s non-leisure-time PA quartiles.

*OR* odds ratio, *PA* physical activity

^a^Adjusted for sex, age, marital status, education, occupation, body mass index, smoking habit, and alcohol consumption

^b^Further adjusted for disease history in addition to covariates in Model 1

^c^Adjusted for sex, age, marital status, education, occupation, body mass index, smoking habit, alcohol consumption, and non-leisure-time PA

^d^Adjusted for sex, age, marital status, education, occupation, body mass index, smoking habit, alcohol consumption, and leisure-time PA

## References

[1] Kamimura S, Iida T, Watanabe Y (2022). Physical activity and recurrent fall risk in community-dwelling Japanese people aged 40–74 years: the Murakami cohort study. Eur Rev Aging Phys Act.

