# Supplementary material for: Physical activity and recurrent fall risk in community-dwelling Japanese people aged 40–74 years: the Murakami cohort study
Source: Eur Rev Aging Phys Act. 2022 Sep 2;19:20. doi: 10.1186/s11556-022-00300-5 (PMC9438326; doi:10.1186/s11556-022-00300-5)
Supplement: Supplementary file 1 — Additional file 1: Table 1. Odds ratios for recurrent falls according to four levels of each physical activity stratified by sex. Table 2. Odds ratios for recurrent falls according to four levels of each physical activity stratified by age group. Table 3. Odds ratios for recurrent falls according to levels of leisure-time PA by non-leisure-time PA subgroup. Table 4. Odds ratios for recurrent falls according to intensity-based levels of leisure-time physical activity. [file 11556_2022_300_MOESM1_ESM.docx]

Additional Table 1. Odds ratios for recurrent falls according to four levels of each physical activity stratified by sex

Quartiles of total PA (MET-hr/day) P for trend

Q1 Q2 Q3 Q4

Men

Cumulative incidence 25/843 (3.0%) 38/874 (4.3%) 44/862 (5.1%) 59/865 (6.8%)

Unadjusted OR (95% CI) 1 (ref) 1.49 (0.89-2.49) 1.76 (1.07-2.90) 2.40 (1.49-3.86) 0.0002

Adjusted OR (1)^a^ (95% CI) 1 (ref) 1.34 (0.79-2.27) 1.49 (0.87-2.54) 2.03 (1.16-3.54) 0.0185

Adjusted OR (2)^b^ (95% CI) 1 (ref) 1.31 (0.77-2.22) 1.50 (0.88-2.58) 2.10 (1.19-3.69) 0.0193

Women

Cumulative incidence 25/998 (2.5%) 31/1,055 (2.9%) 36/1,031 (3.5%) 50/1,033 (4.8%)

Unadjusted OR (95% CI) 1 (ref) 1.18 (0.69-2.01) 1.41 (0.84-2.36) 1.98 (1.22-3.23) 0.0032

Adjusted OR (1)^a^ (95% CI) 1 (ref) 1.20 (0.70-2.07) 1.43 (0.84-2.43) 1.76 (1.04-2.99) 0.0043

Adjusted OR (2)^b^ (95% CI) 1 (ref) 1.22 (0.71-2.10) 1.42 (0.83-2.42) 1.77 (1.04-3.02) 0.0048

Four levels of leisure-time PA (MET-h /day) P for trend

Tertiles for scores > 0

0 Low Medium High

Men

Cumulative incidence 55/1,105 (5.0%) 34/763 (4.5%) 34/767 (4.4%) 43/809 (5.3%)

Unadjusted OR (95% CI) 1 (ref) 0.89 (0.58-1.38) 0.89 (0.57-1.37) 1.07 (0.71-1.61) 0.8205

Adjusted OR (1)^c^ (95% CI) 1 (ref) 1.04 (0.66-1.63) 0.95 (0.59-1.53) 1.21 (0.75-1.93) 0.5987

Adjusted OR (2)^b^ (95% CI) 1 (ref) 1.03 (0.66-1.62) 0.92 (0.57-1.47) 1.20 (0.75-1.93) 0.6874

Women

Cumulative incidence 51/1,320 (3.9%) 32/916 (3.5%) 23/944 (2.4%) 36/937 (3.8%)

Unadjusted OR (95% CI) 1 (ref) 0.90 (0.57-1.41) 0.62 (0.38-1.02) 0.99 (0.64-1.54) 0.5700

Adjusted OR (1)^c^ (95% CI) 1 (ref) 0.85 (0.53-1.35) 0.53 (0.31-0.89) 0.66 (0.40-1.07) 0.0327

Adjusted OR (2)^b^ (95% CI) 1 (ref) 0.83 (0.52-1.32) 0.50 (0.29-0.85) 0.63 (0.39-1.03) 0.0239

Quartiles of non-leisure-time PA (MET-hr/day) P for trend

Q1 Q2 Q3 Q4

Men

Cumulative incidence 26/828 (3.1%) 44/879 (5.0%) 36/869 (4.1%) 60/868 (6.9%)

Unadjusted OR (95% CI) 1 (ref) 1.63 (0.99-2.67) 1.33 (0.80-2.23) 2.29 (1.43-3.67) 0.0016

Adjusted OR (1)^d^ (95% CI) 1 (ref) 1.52 (0.92-2.51) 1.09 (0.63-1.88) 1.86 (1.08-3.21) 0.0573

Adjusted OR (2)^b^ (95% CI) 1 (ref) 1.54 (0.93-2.54) 1.09 (0.63-1.88) 2.14 (1.47-3.11) 0.0630

Women

Cumulative incidence 25/1,024 (2.4%) 31/1,002 (3.1%) 33/1,060 (3.1%) 53/1,031 (5.1%)

Unadjusted OR (95% CI) 1 (ref) 1.28 (0.75-2.18) 1.28 (0.76-2.18) 2.17 (1.34-3.51) 0.0016

Adjusted OR (1)^d^ (95% CI) 1 (ref) 1.37 (0.79-2.35) 1.35 (0.79-2.31) 2.33 (1.39-3.92) 0.0007

Adjusted OR (2)^b^ (95% CI) 1 (ref) 1.36 (0.79-2.34) 1.36 (0.79-2.32) 2.14 (1.47-3.11) 0.0007

OR: odds ratio, PA: physical activity

*Note*: Cut-off values are 37.8, 44.1, and 55.0 for men’s total PA quartiles; 37.8, 42.8, and 51.4 for women’s total PA quartiles; 0.9 and 3.0 for men’s leisure-time PA tertiles; 1.0 and 3.2 for women’s leisure-time PA tertiles; 15.9, 24.2, and 38.6 for men’s non-leisure-time PA quartiles; and 17.7, 24.6, and 36.1 for women’s non-leisure-time PA quartiles.

^a^Adjusted for age, marital status, education, occupation, body mass index, smoking habit, and alcohol consumption

^b^Further adjusted for disease history in addition to covariates in Model 1

^c^Adjusted for age, marital status, education, occupation, body mass index, smoking habit, alcohol consumption, and non-leisure-time PA

^d^Adjusted for age, marital status, education, occupation, body mass index, smoking habit, alcohol consumption, and leisure-time PA

Additional Table 2. Odds ratios for recurrent falls according to four levels of each physical activity stratified by age group

Quartiles of total PA (MET-hr/day) P for trend

Q1 Q2 Q3 Q4

Age <65 years

Cumulative incidence 33/1,379 (2.4%) 44/1,258 (3.5%) 44/1,163 (3.8%) 54/1,116 (4.8%)

Unadjusted OR (95% CI) 1 (ref) 1.48 (0.94-2.34) 1.60 (1.01-2.54) 2.07 (1.34-3.22) 0.0013

Adjusted OR (1)^a^ (95% CI) 1 (ref) 1.43 (0.90-2.28) 1.53 (0.94-2.47) 1.83 (1.11-3.03) 0.0077

Adjusted OR (2)^b^ (95% CI) 1 (ref) 1.47 (0.92-2.35) 1.52 (0.94-2.46) 1.90 (1.15-3.16) 0.0070

Age ≥65 years

Cumulative incidence 17/462 (3.7%) 25/671 (3.7%) 36/730 (4.9%) 55/782 (7.0%)

Unadjusted OR (95% CI) 1 (ref) 1.01 (0.54-1.90) 1.36 (0.75-2.45) 1.98 (1.14-3.46) 0.0024

Adjusted OR (1)^a^ (95% CI) 1 (ref) 1.02 (0.54-1.93) 1.33 (0.73-2.45) 2.07 (1.13-3.77) 0.0101

Adjusted OR (2)^b^ (95% CI) 1 (ref) 1.02 (0.54-1.94) 1.42 (0.76-2.63) 2.18 (1.18-4.00) 0.0085

Four levels of leisure-time PA (MET-h /day) P for trend

Tertiles for scores >0

0 Low Medium High

Age <65 years

Cumulative incidence 74/1,886 (3.9%) 44/1,208 (3.6%) 24/1,039 (2.3%) 33/783 (4.2%)

Unadjusted OR (95% CI) 1 (ref) 0.93 (0.63-1.35) 0.58 (0.36-0.92) 1.08 (0.71-1.64) 0.5022

Adjusted OR (1)^c^ (95% CI) 1 (ref) 0.93 (0.63-1.38) 0.58 (0.35-0.94) 0.88 (0.56-1.40) 0.2294

Adjusted OR (2)^b^ (95% CI) 1 (ref) 0.93 (0.63-1.37) 0.56 (0.34-0.91) 0.86 (0.54-1.37) 0.1902

Age ≥65 years

Cumulative incidence 32/539 (5.9%) 22/471 (4.7%) 33/672 (4.9%) 46/963 (4.8%)

Unadjusted OR (95% CI) 1 (ref) 0.82 (0.50-1.35) 0.80 (0.50-1.26) 0.85 (0.53-1.35) 0.4128

Adjusted OR (1)^c^ (95% CI) 1 (ref) 0.86 (0.51-1.47) 0.90 (0.55-1.48) 0.99 (0.61-1.62) 0.7499

Adjusted OR (2)^b^ (95% CI) 1 (ref) 0.86 (0.50-1.46) 0.93 (0.56-1.52) 0.98 (0.60-1.60) 0.7370

Quartiles of non-leisure-time PA (MET-hr/day) P for trend

Q1 Q2 Q3 Q4

Age <65 years

Cumulative incidence 30/1,229 (2.4%) 43/1,251 (3.4%) 43/1,232 (3.5%) 59/1,204 (4.9%)

Unadjusted OR (95% CI) 1 (ref) 1.42 (0.89-2.28) 1.45 (0.90-2.32) 2.06 (1.32-3.22) 0.0019

Adjusted OR (1)^d^ (95% CI) 1 (ref) 1.39 (0.86-2.25) 1.47 (0.90-2.39) 1.91 (1.16-3.16) 0.0068

Adjusted OR (2)^b^ (95% CI) 1 (ref) 1.38 (0.85-2.23) 1.48 (0.91-2.41) 2.14 (1.47-3.11) 0.0071

Age ≥65 years

Cumulative incidence 21/623 (3.4%) 32/630 (5.1%) 26/697 (3.7%) 54/695 (7.8%)

Unadjusted OR (95% CI) 1 (ref) 1.53 (0.87-2.69) 1.11 (0.62-2.00) 2.42 (1.44-4.05) 0.0018

Adjusted OR (1)^d^ (95% CI) 1 (ref) 1.52 (0.86-2.69) 0.93 (0.51-1.72) 2.60 (1.47-4.62) 0.0074

Adjusted OR (2)^b^ (95% CI) 1 (ref) 1.58 (0.89-2.81) 0.98 (0.53-1.83) 2.14 (1.47-3.11) 0.0057

OR: odds ratio, PA: physical activity

*Note*: Cut-off values are 37.8, 44.1, and 55.0 for men’s total PA quartiles; 37.8, 42.8, and 51.4 for women’s total PA quartiles; 0.9 and 3.0 for men’s leisure-time PA tertiles; 1.0 and 3.2 for women’s leisure-time PA tertiles; 15.9, 24.2, and 38.6 for men’s non-leisure-time PA quartiles; and 17.7, 24.6, and 36.1 for women’s non-leisure-time PA quartiles.

^a^Adjusted for age, sex, marital status, education, occupation, body mass index, smoking habit, and alcohol consumption

^b^Further adjusted for disease history in addition to covariates in Model 1

^c^Adjusted for age, sex, marital status, education, occupation, body mass index, smoking habit, alcohol consumption, and non-leisure-time PA

^d^Adjusted for age, sex, marital status, education, occupation, body mass index, smoking habit, alcohol consumption, and leisure-time PAAdditional Table 3. Odds ratios for recurrent falls according to levels of leisure-time PA by non-leisure-time PA subgroup

Levels of leisure-time PA (MET-h /day) P for trend

Tertiles for scores > 0

0 Low Medium High

Lower levels of non-leisure-time PA (Quartiles 1 and 2)

Cumulative incidence 49/1,130 (4.3%) 23/897 (2.6%) 24/938 (2.6%) 30/768 (3.9%)

Unadjusted OR (95% CI) 1 (ref) 0.58 (0.35-0.96) 0.58 (0.35-0.95) 0.90 (0.56-1.43) 0.4006

Adjusted OR (1)^a^ (95% CI) 1 (ref) 0.54 (0.32-0.91) 0.46 (0.27-0.79) 0.58 (0.34-1.00) 0.0288

Adjusted OR (2)^b^ (95% CI) 1 (ref) 0.55 (0.33-0.93) 0.47 (0.28-0.81) 0.59 (0.35-1.02) 0.0314

Higher levels of non-leisure-time PA (Quartiles 3 and 4)

Cumulative incidence 57/1,295 (4.4%) 43/782 (5.5%) 33/773 (4.3%) 49/978 (5.0%)

Unadjusted OR (95% CI) 1 (ref) 1.26 (0.84-1.90) 0.97 (0.63-1.50) 1.15 (0.78-1.69) 0.7108

Adjusted OR (1)^a^ (95% CI) 1 (ref) 1.38 (0.91-2.10) 1.05 (0.65-1.68) 1.32 (0.83-2.08) 0.5928

Adjusted OR (2)^b^ (95% CI) 1 (ref) 1.32 (0.86-2.02) 0.96 (0.60-1.55) 1.24 (0.78-1.97) 0.7390

PA: physical activity

*Note:* Cut-off values are 0.9 and 3.0 for men’s leisure-time PA tertiles, 1.0 and 3.4 for women’s leisure-time PA tertiles

^a^Adjusted for age, marital status, education, occupation, body mass index, smoking habit, alcohol consumption, and non-leisure-time PA

^b^Further adjusted for disease history in addition to covariates in Model (1)

Additional Table 4. Odds ratios for recurrent falls according to intensity-based levels^a^ of leisure-time physical activity

Intensity levels of leisure-time physical activity

None Low Moderate High

Cumulative incidence 132/3,215 (4.1%) 79/1,834 (4.3%) 83/1,961 (4.2%) 14/551 (2.5%)

Unadjusted OR (95% CI) 1 (ref) 1.05 (0.79-1.40) 1.03 (0.78-1.37) 0.61 (0.35-1.06)

Age-adjusted OR (95% CI) 1 (ref) 1.02 (0.77-1.36) 0.86 (0.64-1.15) 0.57 (0.33-1.01)

Adjusted OR (1)^b^ (95% CI) 1 (ref) 1.14 (0.85-1.53) 0.92 (0.68-1.25) 0.61 (0.34-1.09)

Adjusted OR (2)^c^ (95% CI) 1 (ref) 1.14 (0.85-1.53) 0.93 (0.68-1.26) 0.61 (0.34-1.08)

^a^“None”, 0 min/week for both moderate- and strenuous-intensity exercises; “Low”, moderate-intensity exercise 0-149 and strenuous-intensity exercise hours 0-74 min/week (excluding 0 min/week for both exercises); “Moderate”, moderate-intensity exercise ≥150 and strenuous-intensity exercise <75 min/week; and “High”, strenuous-intensity exercise ≥75 min/week, where “moderate-intensity exercise” includes “walking quickly” and “light to moderate exercise” of JPHC-PAQ.

^b^Adjusted for sex, age, marital status, education, occupation, body mass index, smoking habit, alcohol consumption, and non-leisure-time physical activity

^c^Further adjusted for disease history in addition to covariates in Model (1)
